# Ahnak scaffolds p11/Anxa2 complex and L-type voltage-gated calcium channel and modulates depressive behavior

**DOI:** 10.1038/s41380-019-0371-y

**Published:** 2019-02-13

**Authors:** Junghee Jin, Dionnet L. Bhatti, Ko-Woon Lee, Lucian Medrihan, Jia Cheng, Jing Wei, Ping Zhong, Zhen Yan, Cassandra Kooiker, Claire Song, Jung-Hyuck Ahn, Gerald J. Obermair, Amy Lee, Jodi Gresack, Paul Greengard, Yong Kim

**Affiliations:** 1 Laboratory of Molecular and Cellular Neuroscience, The Rockefeller University, 1230 York Avenue, New York, NY 10065 USA; 2 Department of Physiology and Biophysics, School of Medicine and Biomedical Sciences, State University of New York, Buffalo, NY USA; 3 Department of Biochemistry, Ewha Womans University, Seoul, South Korea; 4 Division of Physiology, Medical University Innsbruck, 6020 Innsbruck, Austria; 5 Department of Molecular Physiology and Biophysics, University of Iowa, Iowa City, IA USA

**Keywords:** Neuroscience, Biochemistry, Depression

## Abstract

Genetic polymorphisms of the L-type voltage-gated calcium channel (VGCC) are associated with psychiatric disorders including major depressive disorder. Alterations of S100A10 (p11) level are also implicated in the etiology of major depressive disorder. However, the existence of an endogenous regulator in the brain regulating p11, L-type VGCC, and depressive behavior has not been known. Here we report that Ahnak, whose function in the brain has been obscure, stabilizes p11 and Anxa2 proteins in the hippocampus and prefrontal cortex in the rodent brain. Protein levels of Ahnak, p11, and Anxa2 are highly and positively correlated in the brain. Together these data suggest the existence of an Ahnak/p11/Anxa2 protein complex. Ahnak is expressed in p11-positive as well as p11-negative neurons. Ahnak, through its N-terminal region, scaffolds the L-type pore-forming α1 subunit and, through its C-terminal region, scaffolds the β subunit of VGCC and the p11/Anxa2 complex. Cell surface expression of the α1 subunits and L-type calcium current are significantly reduced in primary cultures of Ahnak knockout (KO) neurons compared to wild-type controls. A decrease in the L-type calcium influx is observed in both glutamatergic neurons and parvalbumin (PV) GABAergic interneurons of Ahnak KO mice. Constitutive Ahnak KO mice or forebrain glutamatergic neuron-selective Ahnak KO mice display a depression-like behavioral phenotype similar to that of constitutive p11 KO mice. In contrast, PV interneuron-selective Ahnak KO mice display an antidepressant-like behavioral phenotype. Our results demonstrate L-type VGCC as an effector of the Ahnak/p11/Anxa2 complex, revealing a novel molecular connection involved in the control of depressive behavior.

## Introduction

S100A10 (p11) is a member of the S100 protein family [1]. Alterations of p11 are implicated in the etiology of major depressive disorder (MDD) and in the therapeutic actions of antidepressants [2]. The levels of p11 mRNA and protein in the brain are downregulated in depressed humans, suicide victims, and a mouse model of depression [3–5], suggesting an important role for p11 in depression pathophysiology [5]. p11 null mice exhibit depression-like behaviors and abolished behavioral responses to antidepressants [3, 6]. Conversely, p11 overexpression in mice leads to an antidepressant-like behavioral phenotype [3]. Because p11 is an adaptor-like small protein with a molecular weight of 11 kDa, its function is likely mediated by its interacting partners. Thus, it is critical to identify binding partners and downstream effectors of p11 and characterize their role in depression-like behaviors in order to fully understand the mechanism by which p11 controls depression-like behaviors.

In an initial study in our laboratory, p11 was identified as a binding partner of several subtypes of serotonin receptors such as 5HT1B, 5HT1D, and 5HT4 by yeast two-hybrid assays [3, 7]. p11 increases the surface expression of 5HT1B and 5HT4, thereby potentiating serotonergic signaling and facilitating the actions of antidepressants such as selective serotonin reuptake inhibitors (SSRIs) [2, 3, 7]. p11 forms a heterotetrameric protein complex with Anxa2 [8]. We identified a chromatin-remodeling factor, named SMARCA3, as a binding partner of the p11/Anxa2 complex from HEK293 cells [9]. SMARCA3 constitutive knockout (KO) did not cause depression-like behaviors but it abolished behavioral and neurogenic responses to SSRIs [9]. Using this binding assay, we also identified Ahnak as a binding partner of the p11/Anxa2 complex [9]. Ahnak is an extremely large protein with a molecular weight of 680 kDa [10, 11]. The interaction of Ahnak with the p11/Anxa2 protein complex was first demonstrated in a canine kidney cell line Madin-Darby Canine Kidney (MDCK), in which p11 and Anxa2 were required for recruitment of Ahnak to the plasma membrane [12]. Previous reports showed Ahnak expression in endothelial cells in the blood brain barrier, epithelial cells in the choroid plexus and ependymal cells in the ventricular wall of the adult mouse brain [13, 14], in which a role for Ahnak in the formation of tight junctions was proposed [13]. However, the neuronal function of Ahnak and the functional significance of its interaction with the p11/Anxa2 complex in the brain have not yet been investigated.

L-type voltage-gated calcium channels (VGCCs) are heteromultimeric protein complexes composed of a pore-forming α1 subunit and two auxiliary subunits: cytoplasmic β subunit and extracellular α_2_/δ subunit [15]. Two L-type α1 subunits (Ca_v_1.2 and Ca_v_1.3) are expressed in the brain, and L-type VGCCs are mainly localized in the soma and dendrites of neurons. Voltage-dependent L-type channel opening and calcium influx initiate both CaMKIV and MAPK signaling pathways, eventually leading to activation of CREB-dependent gene transcription [16–18]. Because L-type VGCC-mediated calcium signaling and gene regulation contribute to neuronal development, synaptic plasticity, and homeostatic control of neuronal circuitry [19, 20], alterations of the level or activity of L-type VGCC may result in behavioral abnormalities. In fact, previous genome-wide association studies have shown that Ca_v_1.2 gene (CACNA1C) polymorphisms are associated with MDD, bipolar disorder, and schizophrenia [21–23]. Alterations of L-type channels have emerged as susceptibility factors for multiple psychiatric disorders [21, 24, 25]. Intriguingly, Ahnak was known as a binding partner of the β subunit of cardiac VGCC [26, 27] and the regulation of L-type VGCCs by Ahnak has been reported in cardiomyocytes [26–28], osteoblasts [29], and T cells [30], implicating Ahnak in the regulation of neuronal L-type VGCC.

In the present study, we show that Ahnak is a stabilizer of the p11/Anxa2 protein complex and a pivotal regulator of L-type VGCCs in the brain. In addition, our results indicate that neuronal type-selective Ahnak KO mice display modulated depressive behavior, elucidating a neuronal function of Ahnak potentially relevant to the pathophysiology of psychiatric disorders.

## Materials and methods

### Animals

All procedures for biochemical and behavioral experiments involving animals were approved by The Rockefeller University Institutional Animal Care and Use Committee and were in accordance with the National Institutes of Health guidelines. p11 KO mice were generated previously [3]. Ahnak KO mice [31] were received from RIKEN Bio Resource Center. p11-EGFP (HC85) mice were obtained from GENSAT (www.gensat.org). Floxed Ahnak mice were generated in Taconic-Artemis (Germany) and maintained at The Rockefeller University. EMX-Cre (stock 005628) and PV-Cre (stock 008069) lines were obtained from The Jackson Laboratory (Bar Harbor, ME, USA). We produced the progeny of the Ahnak KO lines by in vitro fertilization (IVF) and embryo transfer techniques (Transgenic facility, The Rockefeller University) to provide a sufficient number of animals of the same age for the behavioral tests. All mice are of C57BL/6 background. Sixteen-week-old male mice were used for behavioral tests. 6-8 weeks old male mice were used for whole-cell patch-clamp recordings. Timed-pregnant female mice (E17) were used for primary cortical neuronal cultures. Mice were housed 2–5 per cage with a 12:12-h light/dark cycle and *ad libitum* access to food and water. Mice were assigned to experimental groups based on their genotype. Selection of animal samples out of different experimental groups for electrophysiology and biochemical analyses was performed randomly in a blinded fashion.

### Pulldown assay

GST pulldown assay was performed as described previously [9]. Rat forebrain was homogenized with homogenization buffer (50 mM Tris-HCl, pH 7.5, 150 mM NaCl, and 2 mM MgCl_2_ supplemented with 1% Triton X-100 and a protease inhibitor cocktail (cOmplete, Sigma-Aldrich). The soluble fraction was incubated with GST, GST-p11, or GST-p11/Anxa2 hybrid immobilized on glutathione–agarose beads (GE healthcare). After washing out the unbound proteins, bound proteins were subjected to SDS-PAGE using 4–20% Tris-Glycine gel (Thermo Fisher Scientific, Grand Island, NY, USA). After protein staining with Coomassie Brilliant Blue R-250, the identity of the protein band specifically co-isolated with the p11/Anxa2 hybrid was determined by mass spectrometry (Yale/NIDA Neuroproteomics Center, New Haven, CT, USA).

### Plasmid constructs

Plasmids expressing HA-Ca_v_1.2 (sHA-Ca_v_1.2) [32], HA-Ca_v_1.3 (sHA-Ca_v_1.3a) [33], or β_4b_ subunit (pβA-β_4b_-V5) [34] were reported previously. The cDNAs of the N-terminal region (amino acids 2–498) and repetitive elements in the central region of human Ahnak (amino acids 1068–1579) were obtained from Pet28a-AHNAK-N-HIS-T7 and Pet28a-AHNAK-R-HIS-T7 as reported previously [35] and subcloned into the BamHI-XhoI site of a pAAV-CBA vector. The cDNA of the C-terminal region of mouse Ahnak (amino acids 3921–5656) [36] was cloned into the BamHI-EcoRI site of a pAAV-CBA vector. pAAV-CBA-Ahnak-N-Strep, pAAV-CBA-Ahnak-R-Strep, and pAAV-CBA-Ahnak-C-Strep were confirmed by sequencing.

### Quantitative PCR (qPCR)

Total RNA was extracted from PFC and hippocampus using the RNeasy Mini kit (QIAGEN) according to the manufacturer’s protocol. RNA concentration was measured by a Nanodrop 1000 spectrophotometer (Marshall Scientific, Hampton, New Hampshire, USA). Reverse transcription was performed with 1 μg of total RNA using a High Capacity cDNA Reverse Transcription Kit (Thermo Fisher Scientific, Waltham, MA, USA) according to the manufacturer’s protocol. The qPCR was performed in a 20 μl reaction mixture containing 1 μl (10–50 ng) cDNA, 10 μl SYBR Premix EX Taq (Takara Bio, Kusatsu, Shiga Prefecture, Japan), 0.4 μl Rox reference dye (50× , Takara Bio), and 200 nM of primers for each gene using the 7500 fast real-time PCR system (Thermo Fisher Scientific). The primer sequences were as follows: p11 (forward), 5′-TGGAAACCATGATGCTTACGTT-3′; p11 (reverse), 5′-GAAGCCCACTTTGCCATCTC-3′; AnxA2 (forward), 5′- ATGTCTACTGTCCACGAAATCCT-3′; AnxA2 (reverse), 5′- CGAAGTTGGTGTAGGGTTTGACT-3′; GAPDH (forward), 5′- AGGTCGGTGTGAACGGATTTG-3′; GAPDH (reverse), 5′- TGTAGACCATGTAGTTGAGGTCA -3′. The reaction ran at 95 °C for 30 s, followed by 40 cycles of 95 °C for 3 s and 60 °C for 30 s and a dissociation cycle of 95 °C for 15 s, 60 °C for 60 s and 95 °C for 15 s. All PCRs were performed in triplicates and the specificity of the reaction was detected by melting curve analysis at the dissociation stage. Comparative quantification of each target gene was performed based on cycle threshold normalized to GAPDH using the ΔΔCT method [37].

### Immunoblotting and antibodies

Mouse prefrontal cortex (PFC) or hippocampal tissues were lysed with a lysis buffer (Pierce IP Lysis Buffer, 87788, Thermo Fisher Scientific) supplemented with a protease and phosphatase inhibitor cocktail (78442, Thermo Fisher Scientific). The tissue lysates were homogenized with a Dounce homogenizer (10 strokes) and centrifuged at 800 × *g* for 5 min. Protein levels in the supernatant were measured by the BCA method. The samples were mixed with the standard protein sample buffer, and subjected to SDS-PAGE with 4–20% Novex Tris-Glycine gels (Thermo Fisher Scientific), followed by protein transfer onto a nitrocellulose membrane. Immunoblotting was performed with a standard protocol using the following antibodies: anti-Ahnak (M-DY pAb [38], rabbit polyclonal, 1:50,000 or RU2064, rabbit polyclonal, 1:10,000), anti-p11 (goat polyclonal, AF2377, R&D systems, 1:1,000), anti-Anxa2 (mouse monoclonal, sc-28385, Santa Cruz, 1:1000), anti-Gapdh (mouse monoclonal, MAB374, Millipore Sigma, 1:5,000), anti-Ca_v_1.2 (rabbit polyclonal, AB5156, EMD Millipore, 1:2,500), anti-Ca_v_1.3 (rabbit polyclonal, Ab144 [39], 1:1000), anti-Flag (mouse monoclonal, M2, Sigma, 1:1,000), anti-V5 (mouse monoclonal, SV5-Pk1, Abcam, 1:1000), anti-HA (rat monoclonal, 3F10, Sigma, 1:1,000), anti-β_1_ subunit (mouse monoclonal, 73–052, NeuroMab, 1:1000), anti-β_2a_ subunit (rabbit polyclonal antibody, RU2077, 1:1,000), anti-β_3_ subunit (rabbit polyclonal antibody, RU2080, 1:1,000), anti-β_4_ subunit (mouse monoclonal, 75–054, NeuroMab, 1:1000), anti-α_2_δ1 subunit (mouse monoclonal, DCABH-8461, Creative Diagnostics, 1:1000). RU2064, RU2077, and RU2080 sera were generated against antigen peptides (KISMPDVDLHLKGPK [14, 40], CDSETQESRDSAYVEPKEDY [41], and CDRNWQRNRPWPKDSY [41], respectively) (Cocalico Biologicals Inc, PA, USA). Antibodies were purified using affinity column chromatography.

### Tissue culture, transfection, and immunoprecipitation

COS-7 cells (ATCC) were maintained in DMEM medium (Invitrogen) containing 10% fetal bovine serum (Sigma) and antibiotics (penicillin/streptomycin, Thermo Fisher Scientific). Transient transfection of plasmids was done with Lipofectamine 2000 (Invitrogen) according to the manufacturer’s instructions. For immunoprecipitation, cells (or mouse forebrain tissue) were lysed by sonication in homogenization buffer (plus supplements of protease inhibitors and 1% Triton X-100). After centrifugation at 13,000 × *g* for 10 min, the supernatant was incubated with anti-Ahnak antibody, control antibody-coupled A/G agarose beads (Pierce), or Strep-Tactin beads (IBA Lifesciences) overnight at 4 °C with constant rotation. After washing four times with homogenization buffer containing 1% Triton X-100, bound proteins were eluted in the SDS sample buffer. Samples were subjected to SDS-PAGE with 4–20% Tris-Glycine gels (Thermo Fisher Scientific) and immunoblot analysis as indicated.

### Immunohistochemistry

Animals were deeply anesthetized using CO_2_ and transcardially perfused with PBS, followed by 4% paraformaldehyde (PFA) in PBS. Brains were post-fixed in 4% PFA overnight at 4 °C, and then cryoprotected using 30% sucrose in PBS for at least 24 h, followed by freezing and embedding in Tissue Tek OCT medium (Sakura Finetek USA Inc, Torrance, CA)​. A cryostat was used to collect 40-μm-thick coronal sections. All staining between groups used the same master solution mix of blocking buffer and antibodies. Immunohistochemistry was performed side by side between groups. Free-floating sections were washed in PBS and subsequently incubated in blocking buffer (0.5% Triton X-100, 5% normal goat serum, in PBS) for ~2 h at room temperature. Sections were then incubated overnight (~16 h) at 4 °C in the primary antibodies diluted in blocking buffer. The primary antibodies were as follows: anti-EGFP (chicken polyclonal, Abcam, 1:200), anti-Ahnak (rabbit polyclonal, RU2064, 1:1,000), anti-parvalbumin (mouse monoclonal, Swant, 1:1,000). After incubation, sections were washed three times in PBS and incubated with Alexa-fluor-conjugated secondary antibodies. The secondary antibodies were as follows: goat anti-rabbit Alexa Fluor 568 (Invitrogen, 1:1,000), goat anti-chicken or anti-mouse Alexa Fluor 488 (Invitrogen, 1:1,000), and NeuroTrace 435/455 Nissl (1:400, Thermo Fisher Scientific). After secondary incubation, sections were washed in PBS three times and mounted on glass slides with hard set Vectashield (Vector Labs) for microscopy. Confocal images were obtained on a Zeiss LSM 710 confocal imaging system (Carl Zeiss Microscopy, Thornwood, NY, USA) using a 20× /0.8 N.A. air or a 100× /1.4 N.A. oil-immersion objectives (Carl Zeiss Microscopy, Thornwood, NY, USA). Images (20×) were tiled into one large image when appropriate. Sequential scanning was performed as this prevents artifacts due to cross-excitation of the fluorophores of other laser lines and “bleed-through” of fluorophore emission into channels. Multichannel configuration with sequential scanning for three wavelengths were individually excited by 405-/488-/561-nm laser lines and recorded with corresponding wavelength detection range at 410–483 nm, 497–574 nm, and 583–650 nm, respectively. Gain, exposure time, and all other related settings were constant throughout each experiment. Post-processing consisted of importing to Fiji, assigning colors for each channel in the image, and merging the channels to create one image. All image groups were processed in parallel using Fiji.

### Primary cortical neuronal culture and cell surface biotinylation

Primary cortical neurons were prepared as described previously [42] from embryonic brains (embryonic day 16 or 17) of WT and Ahnak homozygote KO mice. A total of 2 × 10^6^ cortical neurons were plated on 35-mm dishes precoated with poly-l-lysine. Neurons were grown in neurobasal medium supplemented with 0.5 mM l-glutamine, B27 (2%), and N2 (1%). 5-Fluoro-2-deoxyuridine (30 μM, Sigma-Aldrich) was added to inhibit proliferation of non-neuronal cells.

On DIV 7, the culture dishes were placed on ice and washed twice with cold PBS + buffer (phosphate buffered saline plus 0.1 mM CaCl_2_, 1 mM MgCl_2_, pH 8.0). Fresh Sulfo-NHS-SS-Biotin (21335, 0.5  mg/ml; Thermo Fisher Fisher) in 1 ml of PBS + buffer was added to the cells. Dishes were gently shaken in the cold room for 30 min on ice, followed by a 5-min wash of cells three times with 100 mM glycine in PBS + on ice and another 5-min wash of cells three times with PBS + on ice in the cold room with gentle shaking. After removal of washing buffer, the neurons were scraped with RIPA lysis buffer (89901, Thermo Fisher Scientific) supplemented with protease and phosphatase inhibitors (78442, Thermo Fisher Scientific) and placed on ice for 10 min. After centrifugation at 16,000 × *g* for 10 min at 4 ^o^C, the supernatant was collected and protein level was measured by BCA assay. Ten micrograms of loading control and an equal amount of total protein (100 µg) were incubated with 20 µl of streptavidin beads (SA10004; Thermo Fisher Scientific). After incubation with rotation at 4 ^o^C overnight, the beads were washed with RIPA buffer four times and the bound proteins were subjected to SDS-PAGE and immunoblotting.

### Whole-cell patch clamping

Mice aged 6–8 weeks were euthanized. We decided to use young animals for the whole-cell patch-clamp recordings because of difficulty in neuronal recording, especially for PV neurons, from slices of 4-month-old animals. The purpose of these experiments was to investigate the neuronal function of Ahnak rather than to make a correlation with the behavioral experiments. For the patch-clamp experiments, we recorded from one WT mouse and one KO mouse as littermates each day. After decapitation and removal of the brains, transverse slices were cut using a Vibratome 1000 Plus (Leica Microsystems, USA). The cutting solution for prefrontal cortex was (in mM): 234 Sucrose, 2.5 KCl, 1.0 NaH_2_PO_4_, 11 Glucose, 4 MgSO_4_, 0.1 CaCl_2_, 15 HEPES (pH ~7.4, 295–305 mOsm). After cutting, slices were left to recover for 1 h at room temperature in ACSF solution. The cutting solution for hippocampus was (in mM): 105 NMDG (N-Methyl-D-glucamine), 105 HCl, 2.5 KCl, 1.2 NaH_2_PO_4_, 26 NaHCO_3_, 25 Glucose, 10 MgSO_4_, 0.5 CaCl_2_, 5 L-Ascorbic Acid, 3 Sodium Pyruvate, 2 Thiourea (pH ~7.4, 295–305 mOsm). After cutting, slices were left to recover for 15 min in the same cutting solution at 35 °C and for 1 h at room temperature in ACSF solution.

Whole-cell patch-clamp recordings of voltage-dependent Ca^2+^-currents were measured with a Multiclamp 700B/Digidata1550A system (Molecular Devices, Sunnyvale CA, USA). The slice was placed in a recording chamber (RC-27L, Warner Instruments, USA) and constantly perfused with oxygenated ACSF at 24 °C (TC-324B, Warner Instruments, USA) at a rate of 1.5–2.0 ml/min. Cells were visualized with an upright Olympus BX51WI microscope (Olympus, Japan). Voltage-dependent Ca^2+^ current was elicited with depolarizing voltage steps from −70 to −10 mV. Recording pipettes (King Precision Glass, Inc, Glass type 8250) were pulled with a horizontal pipette puller (Narishige) to a resistance of 3–4 MΩ.

For the voltage-dependent Ca^2+^ current recording of layer 2/3 pyramidal neurons in medial prefrontal cortical slices (300 µm), the extracellular solution (modified ACSF) contained (in mM): 120 NaCl, 20 NaHCO_3_, 3.0 KCl, 1.25 NaH_2_PO_4_, 20 CsCl, 1.5 CaCl_2_, 5 MgCl_2_, 8 glucose, pH 7.4, and 300 mOsm (bubbled with 95% O_2_ and 5% CO_2_). Tetrodotoxin (1 µM) was added to block Na^+^ currents. The internal solution contained (in mM): 130 Cs-methanesulfonate, 10 CsCl, 4 NaCl, 1 MgCl_2_, 5 EGTA, 10 HEPES, 5 MgATP, 0.5 Na_3_GTP, 12 phosphocreatine, 3 lidocaine, pH 7.2–7.3, and 265–270 mOsM.

For the recording of parvalbumin (PV) neurons from hippocampal slices (400 μm), the extracellular solution (ACSF) contained (in mM): 125 NaCl, 25 NaHCO_3_, 2.5 KCl, 1.25 NaH_2_PO_4_, 2 CaCl_2_, 1 MgCl_2_, and 25 glucose (bubbled with 95% O_2_ and 5% CO_2_). Tetradotoxin (1 µM) was added to the extracellular solution. The internal solution contained (in mM): 110 CsCl_2_, 30 TEA-Cl, 1 CaCl_2_, 10 EGTA, 2 MgCl_2_, 4 Na_3_ATP, 0.5 Na_3_GTP, and 10 HEPES, pH 7.3. PV neurons were selected for recording based on their size, shape, and position in the subgranular layer.

For the voltage-dependent Ca^2+^-current recording of primary cortical neurons, neuronal cultures were prepared from brain tissue dissected from 17-day mouse embryos. Whole-cell patch-clamp recording was performed in cultured neurons on DIV 11–12. Extracellular ACSF solution and internal solution were the same as those used for recording of PV neurons in brain slices. Triangle-shaped pyramidal cells were selected for recording. Cells with no calcium current response were excluded from statistical analysis.

Data were acquired at a sampling frequency of 50 kHz, filtered at 1 kHz and analyzed offline using pClamp10 software (Molecular Devices, Sunnyvale, CA). All electrophysiological data are expressed as means ± SEM. Statistical analysis was performed using the two-tailed unpaired Student’s *t* test.

### Behavioral tests

All behavioral tests were performed during the light cycle. Behavioral assays were run by an experimenter who was blinded to the genotype group allocation. Four-month-old naïve cohorts of WT and constitutive Ahnak KO mice were used for each behavioral assay. Four-month-old naïve cohorts of EMX-KO or PV-KO mice and their control mice were used for open field test (OFT) and depression-like behavioral tests in the following order: OFT (day 1), sucrose preference test (SPT; days 2–4), forced swim test (FST; day 5) and tail suspension test (TST; day 6). In our previous studies with p11 KO and SMARCA3 KO lines, depression-like behavioral assays were performed in adulthood (~3–4 months) [9, 43]. In order to compare behavioral data from Ahnak KO lines to the results obtained in our previous studies of p11 and SMARCA3, we performed behavioral assays at a similar age. Eight open field boxes, four FST tanks or four TST units were used at the same time. Mice from each group were evenly assigned to each equipment (e.g. boxes or tanks) side by side within each run. Equipment assignment for experimental groups was counterbalanced across runs. FST and TST were carried out as described previously [3, 44]. An automated TST/FST device (Clever Sys Inc, Reston, VA, USA) measured the duration of behavioral immobility, which was scored and analyzed during the last 4 min of a 6-min trial. Accuscan (Omnitech Electronics, Columbus, OH) open field equipment and software were used to calculate total distance traveled. SPT was performed as described previously [9] with some modifications [43]. The mice were given a choice of two water bottles for a 1-day habituation period and then one water bottle was replaced with a bottle containing 1.5% sucrose solution. The consumption of water and sucrose solution was measured after 24 h. The sucrose preference was calculated as the ratio of consumed sucrose solution to consumed water. Mice included in the data analysis were in good health throughout behavioral testing. Statistical outliers, defined as scores 2 standard deviations above or below the group mean in each behavioral test, were excluded prior to statistical data analysis.

### Statistical analyses

For all quantitative data for protein or mRNA levels, electrophysiology, and behavioral tests, *n* is the number of biological replicates, and the bar graphs are representative of two or three independent experiments. To determine the numbers of animals per group (for biochemical, electrophysiology, and behavioral experiments), calculations were based on empirical data accumulated. The number of animals for each experiment was appropriate to detect biochemical and behavioral differences. Differences between groups were assessed using unpaired two-tailed parametric *t* test. The behavioral data passed the D’Agostino–Pearson normality test and Shapiro–Wilk normality test. The cell number per group for electrophysiology experiments is based on empirical data accumulated in the laboratory. For the patch-clamp data, we used the two-tailed parametric *t* test, which was preceded by a D’Agostino-Pearson normality test to confirm normal distribution of the data. The variance between the groups was similar. All data are presented as means ± SEM, and *P* values less than 0.05 were defined as statistically significant. All analyses were carried out using GraphPad Prism, version 7.04 (GraphPad Software, San Diego, CA, USA).

## Results

### Ahnak, as a major binding partner of the p11/Anxa2 complex, stabilizes p11 and Anxa2 proteins in the brain

**Fig. 1:**
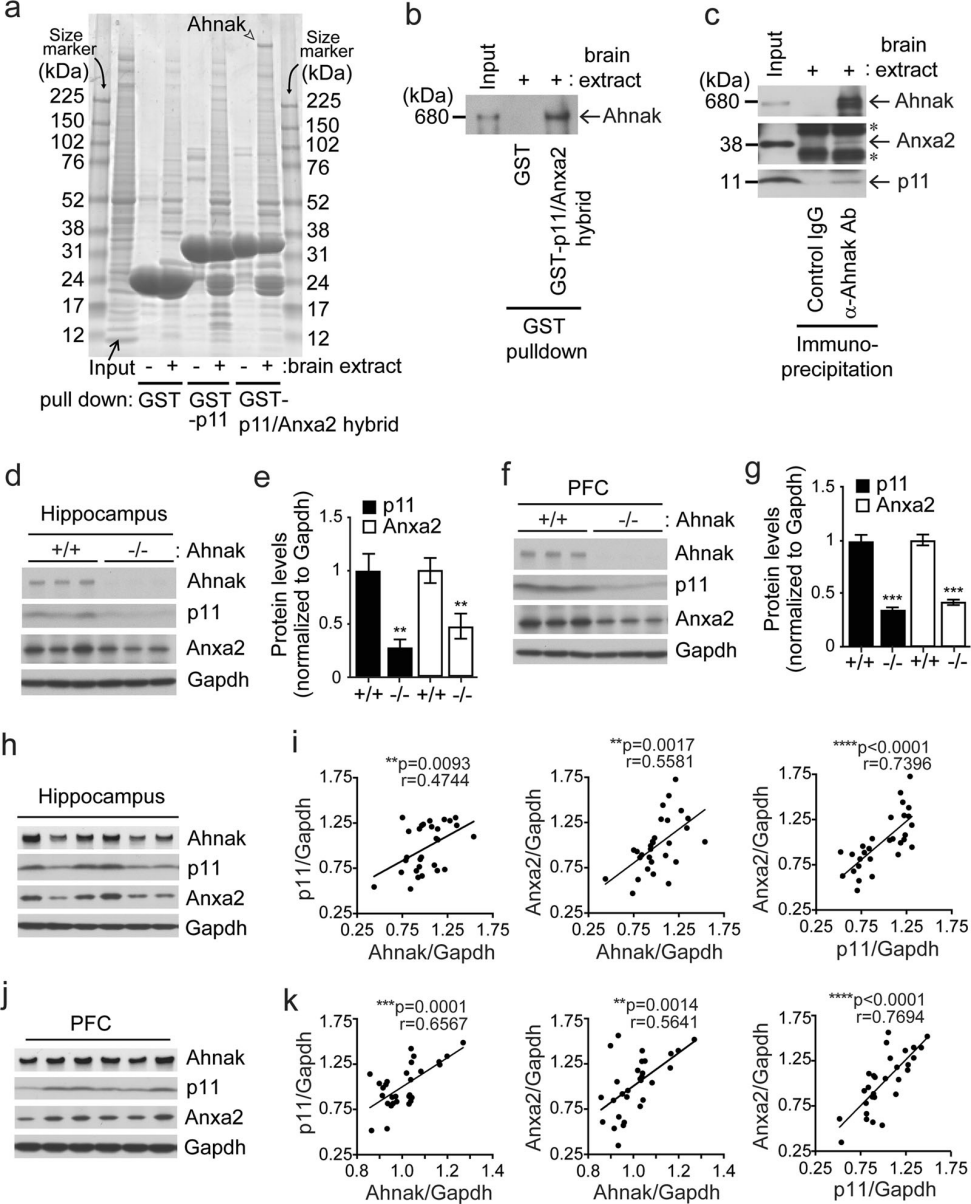
Ahnak, as a major binding partner of the p11/Anxa2 complex, stabilizes p11 and Anxa2 proteins. **a** GST, GST-p11, or GST-p11/Anxa2 peptide hybrid was incubated without (−) or with ( + ) detergent extract of rat brain. After precipitation of GST proteins by glutathione-agarose, total brain extract protein (Input) and co-precipitated proteins were analyzed in SDS-PAGE. A major protein selectively co-precipitated with GST-p11/Anxa2 peptide hybrid (arrowhead) was identified as Ahnak by mass spectrometry. **b** Co-precipitation of Ahnak in the GST pulldown assay was validated by immunoblotting with anti-Ahnak antibody. **c** A detergent-soluble fraction from mouse brain was used for immunoprecipitation with control IgG or anti-Ahnak antibody. p11 and Anxa2 were co-precipitated with Ahnak. Asterisks indicate IgG. **d**-**g** Protein levels of p11 and Anxa2 in the hippocampus (**d**, **e**) or PFC (**f, g**) of WT and Ahnak KO mice were analyzed. Representative images of immunoblotting (**d**, **f**) and quantification of protein levels (**e**, **g**) are shown (*n* = 7 per group). Bars are means ± SEM. **p < 0.01, ***p < 0.001, *t* test. **h**–**k** Comparison of protein levels of Ahnak, p11, and Anxa2 in the brain. Representative images of immunoblotting (**h**, **j**) and comparison plot for Ahnak versus p11, Ahnak versus Anxa2, and p11 versus Anxa2 (**i**, **k**) in the hippocampus (**h**, **i**) or PFC (**j**, **k**) of naïve WT mice. Protein levels of Ahnak, p11, and Anxa2 are positively correlated in an individual brain tissue (*n* = 29). XY comparisons. Pearson’s correlation coefficient, r

In an effort to search for binding partners of the p11/Anxa2 complex in the brain, a protein extract from the rat brain was incubated with immobilized GST, GST-p11, or GST-p11/Anxa2 hybrid, a fusion protein in which the N-terminal 15 amino acids of Anxa2 were fused to p11 with a nine-amino-acid linker [45] (Fig. 1a). We observed that the p11/Anxa2 hybrid mimics the property of the heterotetrameric holocomplex in formation of a hydrophobic pocket for the binding to its effector proteins such as SMARCA3 and Ahnak [9]. After precipitation of GST proteins with glutathione-coupled agarose beads, the co-precipitated proteins were analyzed in SDS-PAGE. We observed a very high molecular weight protein band as a major protein selectively co-precipitated with GST-p11/Anxa2 hybrid but not with GST or GST-p11 (Fig. 1a, indicated by arrowhead). The protein band was subjected to tryptic digestion, and the generated peptides were analyzed by mass spectrometry. We determined the protein identity as Ahnak (or Ahnak1) by detecting 150 peptides covering 26.5% of the rat Ahnak protein sequence. We also confirmed the co-precipitation of Ahnak with GST-p11/Anxa2 hybrid from mouse brain lysates by immunoblotting (Fig. 1b). In addition, immunoprecipitation of Ahnak from brain extracts co-precipitated p11 and Anxa2 (Fig. 1c). Together, these results indicate that Ahnak is a major binding partner of the p11/Anxa2 complex in the brain.

The components of the p11/Anxa2 protein complex stabilize each other in cells. A previous study showed reduced protein levels of p11 in various tissues from Anxa2 KO mice [46]. The absence of Anxa2 caused p11 degradation in a proteasome-dependent manner, suggesting a role for Anxa2 interaction in the stabilization of p11 [46]. Conversely, the protein level of Anxa2 was also drastically reduced without alteration of its mRNA level in brains of p11 KO mice [9], supporting the physiological relevance of the interaction between the components of the p11/Anxa2 protein complex. In this study, we observed that the protein levels of p11 and Anxa2 were drastically reduced in the hippocampus (Fig. 1d and e) and PFC (Fig. 1f and g) of Ahnak KO mice compared to their wild-type (WT) controls. However, the mRNA levels of p11 and Anxa2 were not altered in the Ahnak KO mice (Supplementary Figure 1a and b). We also observed a drastic decrease of p11 and Anxa2 proteins in primary cultured cortical neurons derived from Ahnak KO embryos (Supplementary Figure 1c). In our previous study, KO of SMARCA3, another binding partner of the p11/Anxa2 complex, did not alter protein levels of p11 and Anxa2 [9]. In addition, protein levels of Ahnak were not altered in the hippocampus and PFC of p11 KO mice (Supplementary Figure 1d and e), suggesting that Ahnak protein is stable in the absence of p11. In the line of Ahnak’s stabilization effect, we observed that protein levels of Ahnak, p11, and Anxa2 in the hippocampus (Fig. 1h and i) and PFC (Figure j and k) are highly and positively correlated. Altogether, our data showing a role of Ahnak in the stabilization of p11 and Anxa2 proteins and positive correlation in the levels of Ahnak, p11 and Anxa2 support the existence of the Ahnak/p11/Anxa2 complex in the brain.

### Ahnak is expressed in neurons as well as blood vessels in the brain

**Fig. 2:**
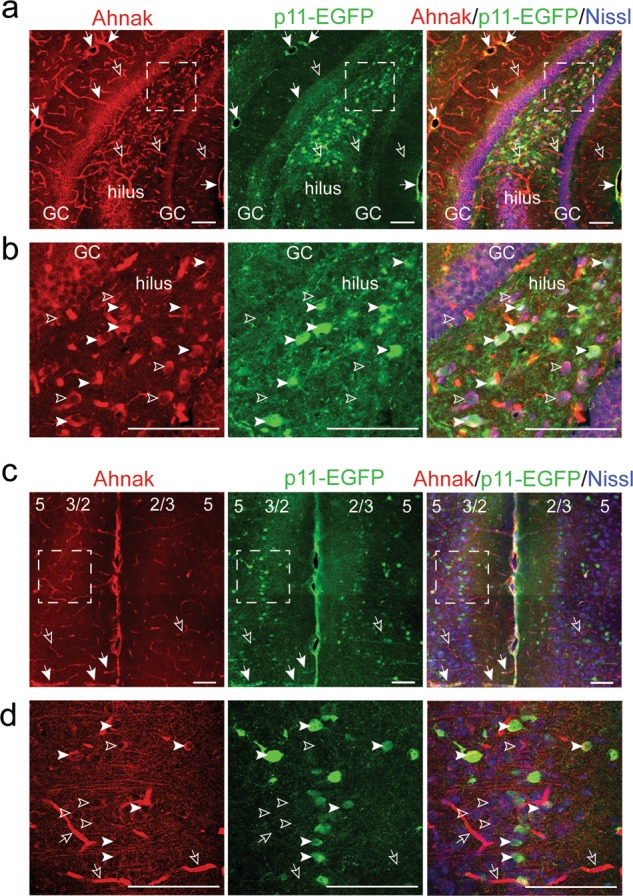
Ahnak is expressed in p11-positive and p11-negative neurons and blood vessels in the hippocampus and PFC. **a**–**d** Immunohistochemistry reveals that Ahnak is expressed ubiquitously in neurons and blood vessels in the DG (**a**, **b**) and PFC (**c**, **d**). p11-expressing cells were examined in the ventral DG of BAC transgenic mice expressing p11 promoter-driven EGFP. Confocal microscopy reveals the expression of Ahnak in p11-positive cells in both the DG (**a**, **b**) and PFC (**c**, **d**). This colocalization is shown at high magnification of the dotted rectangular regions in **a** and **c** (**b**, **d**). Arrows with filled heads indicate Ahnak/p11 colocalization in vessels; arrows with unfilled heads indicate Ahnak in vessels that are p11-negative. p11/Ahnak colocalization in neurons is indicated by filled arrow heads, while Ahnak in neurons that are p11-negative is indicated by unfilled arrow heads. GC, granule cell layer. Cortical layers are indicated in **c**. Nissl counterstaining was used to visualize cellular patterns. Scale bars, 100 µm

A previous immunohistochemistry study of Ahnak in the brain revealed its expression in endothelial cells in the blood–brain barrier and epithelial cells in the choroid plexus, in which Ahnak was co-localized with tight junction marker proteins [13]. In the present study, we also observed the expression of Ahnak in small, intermediate, and large vessels in the dentate gyrus (DG) and PFC by immunohistochemistry with a specific anti-Ahnak antibody (indicated by arrows in Fig. 2a, c, d; Supplementary Figure 2). p11 is expressed in various types of projection neurons and interneurons as well as some non-neuronal cells in the brain [47]. Because our previous studies demonstrated p11 expression in hilar mossy cells and PV-positive interneurons in the DG [9] and pyramidal neurons in layer 2/3 of the PFC [48], we examined possible co-expression of Ahnak in p11-positive neurons in those brain regions. We took advantage of bacterial artificial chromosome (BAC) transgenic mice, in which the expression of EGFP reporter is driven by the p11 promoter (p11-EGFP mice) [9, 47]. Our previous studies of p11-EGFP mice validated that the EGFP-positive cells in the mice represent p11-expressing cells accurately. Compared to the levels of Ahnak in the vessels, a lower but significant expression level of Ahnak was detected in p11-positive neurons (indicated by filled arrow heads) as well as p11-negative neurons (indicated by unfilled arrow heads) in the hilus region of the DG (Fig. 2b) and in layer 2/3 of the PFC (Fig. 2d). p11 promoter-driven EGFP signal was mainly detected in neurons (Fig. 2b and d), but it was also detectable in some of the large or intermediate vascular structures (filled arrows in Fig. 2a and c) but not in most microvessels. These results indicate that Ahnak is expressed in both p11-positive and p11-negative neurons as well as in vascular structures in the brain.

### Ahnak regulates L-type VGCCs in neurons

**Fig. 3:**
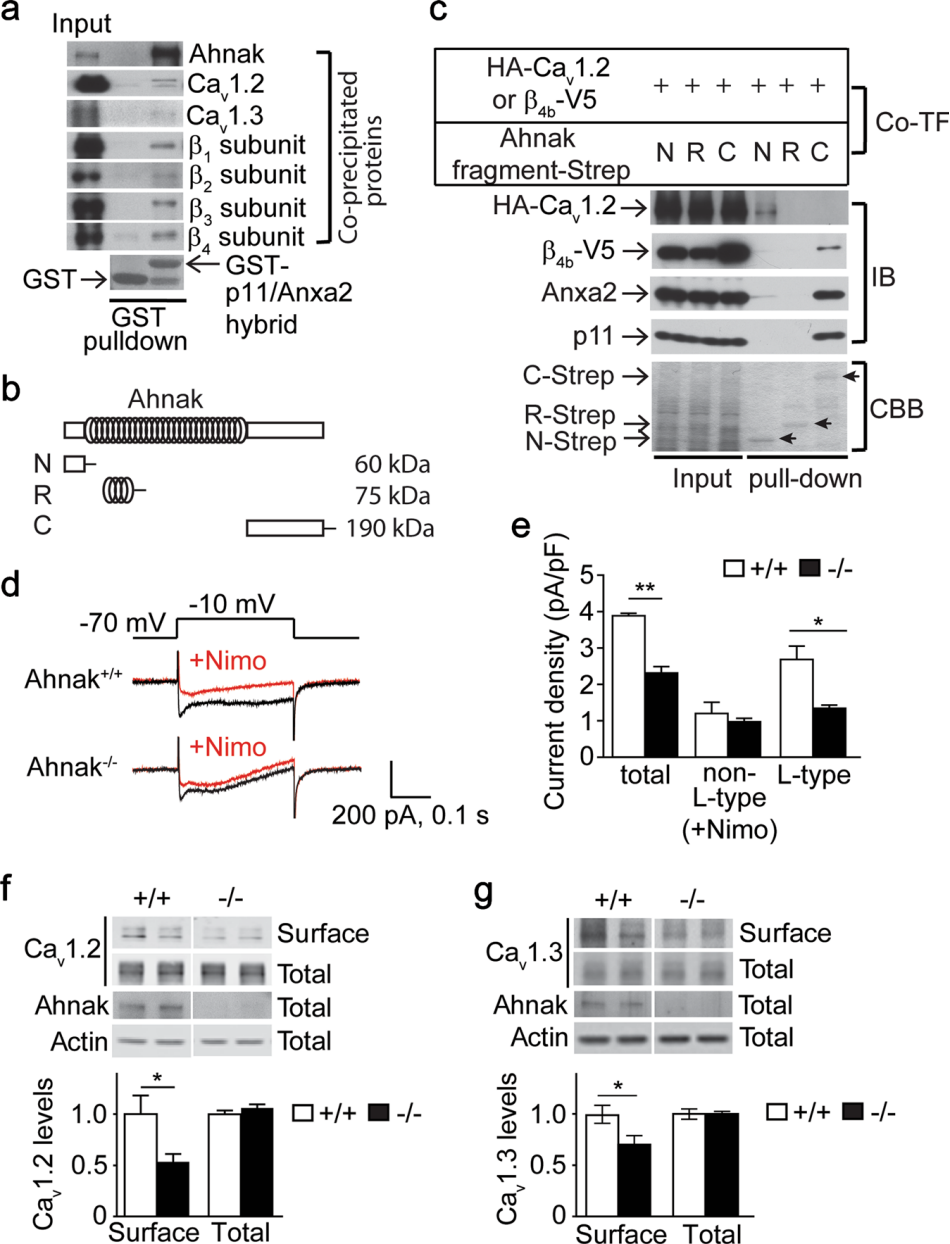
Ahnak interacts with and regulates cell surface expression of α1 subunits of L-type VGCCs. **a** GST or GST-p11/Anxa2 peptide hybrid was incubated with detergent extract of mouse hippocampus. After precipitation of GST proteins by glutathione-agarose, proteins in brain extract (input) and co-precipitated proteins were analyzed for subunits of L-type VGCCs by immunoblotting. Precipitated GST proteins were stained with ponceau S (bottom). **b** Illustration of full length of Ahnak, N-terminal fragment (N), repetitive elements in the central region (R), and C-terminal fragment (C). Estimated molecular mass of the fragments in SDS-PAGE is shown. **c** COS-7 cells were co-transfected with a plasmid expressing HA-tagged Ca_v_1.2 or V5-tagged β4b subunit together with a plasmid expressing Strep-tagged Ahnak fragment of N, R, or C. After pulldown of Strep-tagged Ahnak fragment with Strep-Tactin beads, co-precipitated HA-Ca_v_1.2 or β_4b_-V5 subunit and endogenous p11 and Anxa2 were detected by immunoblotting as indicated. Immunoblotting (IB) images of HA-Ca_v_1.2 or β_4b_-V5 subunit are from HA-Ca_v_1.2- or β_4b_-V5 subunit-transfected cells, respectively. IB of endogenous p11 and Anxa2 and Coomassie brilliant blue (CBB) stain of Ahnak fragments are representative images. **d**-**g** Voltage-induced L-type calcium current and surface expression of Ca_v_1.2 and Ca_v_1.3 are significantly reduced in primary cultured Ahnak KO neurons. Primary cortical neurons were prepared from WT or Ahnak KO embryos. Whole-cell patch-clamp recordings of somatic L-type Ca^2+^ currents were performed on DIV 11–12 before (black) and after (red) the bath addition of nimodipine (10 μM) (**d**, **e**). Representative traces (**d**). Histograms showing the current density of the somatic Ca^2+^ currents (*n* = 5 cells per group) (**e**). **f**, **g** Cell surface proteins were biotinylated and precipitated with streptavidin-coupled beads. Total Ca_v_1.2 (**f**), Ca_v_1.3 (**g**), and actin in lysates and the precipitated surface Ca_v_1.2 (**f**) and Ca_v_1.3 (**g**) were detected by immunoblotting. Quantification of surface level (the surface level normalized to total level) of Ca_v_1.2 or Ca_v_1.3 and total level (total Ca_v_1.2 or Ca_v_1.3 level normalized to total actin) (*n* = 5 per group). Bar graphs are means ± SEM. *p < 0.05, **p < 0.01, *t* test

Ahnak is known to interact with the auxiliary β subunit of cardiac L-type calcium channels at the plasma membrane [26, 49]. In cardiomyocytes, Ahnak is phosphorylated by PKA and modulates Ca_v_1.2 channel activity in response to β-adrenergic receptor stimulation [26, 49]. In T cells, Ahnak is required for L-type Ca_v_1.1-mediated calcium signaling after T-cell receptor activation [30, 50]. Thus, we examined the possible association of L-type VGCCs with the Ahnak/p11/Anxa2 complex in the brain. Protein extract of mouse hippocampus was incubated with immobilized GST or GST-p11/Anxa2 hybrid. After precipitation of GST proteins with glutathione-agarose, the co-precipitated proteins were analyzed by immunoblotting. Two α1 subunits (Ca_v_1.2 and Ca_v_1.3) and all four cytoplasmic β subunits were co-precipitated with Ahnak by GST-p11/Anxa2 hybrid-immobilized beads but not by GST-immobilized beads (Fig. 3a), suggesting that L-type VGCCs interact with the Ahnak/p11/Anxa2 complex.

To clarify the interaction partners among components of the two protein complexes, first we transiently co-transfected an L-type α1 subunit (Ca_v_1.2) or a β subunit (β_4b_) together with Ahnak fragments in COS-7 cells. Because the Ahnak protein is too large to be transiently expressed as a full protein, we used plasmids expressing Strep-tagged N-terminal fragment (N), repetitive elements in the central region (R), or C-terminal fragment (C) of Ahnak (Fig. 3b). Importantly, we observed that pull-down of Strep-tagged N-terminal fragment of Ahnak co-precipitated HA-Ca_v_1.2 (Fig. 3c). In contrast, pull-down of Strep-tagged C-terminal fragment of Ahnak co-precipitated β_4b_ subunit and endogenous p11 and Anxa2 (Fig. 3c), which is consistent with previous reports for the interaction of the C-terminal region of Ahnak with p11/Anxa2 [12] and β_2_ subunit [28, 51]. These results suggest that Ahnak is a scaffolding protein interacting with the L-type α1 subunit through its N-terminal region as well as the β subunit and the p11/Anxa2 complex through its C-terminal region.

We next examined whether Ahnak is necessary for L-type VGCC function. We prepared primary cultures of pure cortical neurons from WT embryos and Ahnak KO (−/−) embryos. Using whole-cell patch-clamp recording, we found that the total voltage-induced calcium current was significantly reduced in Ahnak KO neurons when compared to WT neurons (Fig. 3d and e). We also measured the L-type-specific calcium current and non-L-type calcium current by recording voltage-gated calcium currents in the absence or presence of an L-type-specific inhibitor, nimodipine, or nifedipine [52]. L-type-specific calcium current, but not non-L-type calcium current, was selectively reduced in Ahnak KO neurons when compared to WT neurons (Fig. 3d and e). No significance was observed in membrane capacitance (Cm) between WT neurons and Ahnak KO neurons (Cm (pF): WT, 50.75 ± 12.87 and KO, 44.60 ± 1.72). The cytoplasmic auxiliary β subunit plays a critical role in the modulation of channel activity as well as the membrane trafficking of α1 subunit to the cell surface [34, 53, 54]. In addition, p11 and Anxa2 are required for the surface recruitment of Ahnak to the cell membrane [12]. Thus, we then investigated a possible alteration of the cell surface level of L-type α1 subunits in Ahnak KO neurons. Using a biotinylation assay of cell surface proteins, we observed a decrease of the cell surface levels of Ca_v_1.2 and Ca_v_1.3, but no alteration in their total levels, in Ahnak KO neurons compared to WT controls (Fig. 3f and g). These results indicate a role for Ahnak in the recruitment of Ca_v_1.2 and Ca_v_1.3 to the cell surface and suggest a mechanism by which Ahnak deletion causes a decrease of voltage-induced L-type calcium influx in neurons.

### Ahnak KO causes a decrease of voltage-gated L-type calcium influx in glutamatergic neurons and PV-positive GABAergic interneurons

**Fig. 4:**
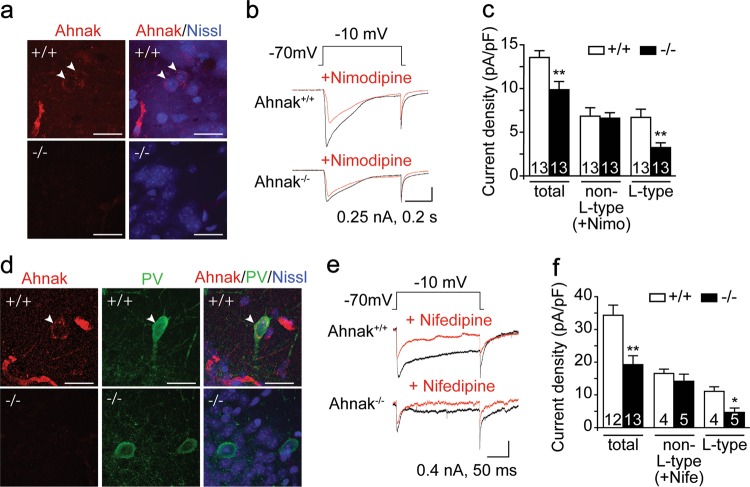
Voltage-induced L-type calcium influx is reduced in glutamatergic neurons and parvalbumin (PV)-positive GABAergic neurons in Ahnak KO mice. Whole-cell patch-clamp recordings of voltage-dependent Ca^2+^ current in pyramidal neurons in layer 2/3 of PFC (**a–c**) or PV-positive GABAergic interneurons in the DG (**d–f**) from WT and Ahnak KO mice. Representative immunostaining images of Ahnak (**a**) or Ahnak and PV (**d**) in WT and Ahnak KO neurons. Scale bars, 25 µm. Voltage-gated somatic Ca^2+^ currents before and after the bath application of an L-type calcium current blocker (nimodipine or nifedipine, 10 μM) (**b**, **c, e,** and **f**). Representative traces (**b**, **e**). Histograms showing the density of total, L-type and non-L-type voltage-gated Ca^2+^ current in neurons from WT and Ahnak KO mice (**c**, **f**). Bar graphs are means ± SEM. *p < 0.05, **p < 0.01, student’s *t* test. The numbers of neurons used for recording are indicated in each bar. 3–5 mice per group

To examine a possible role for Ahnak in the regulation of L-type VGCCs in the brain, we performed whole-cell patch-clamp recordings of pyramidal neurons in layer 2/3 of PFC (Fig. 4a-c) or PV-expressing GABAergic interneurons in the DG (Fig. 4d-f) in WT and Ahnak KO mice. We found that the total voltage-induced calcium current was significantly reduced by Ahnak KO compared to WT controls both in pyramidal neurons in layer 2/3 of PFC (Fig. 4b, c) and PV GABAergic interneurons in the DG (Fig. 4e, f). The reduction of voltage-induced total calcium influx was due to the reduction of L-type-specific calcium current but not non-L-type calcium current in both cell types (Fig. 4b, c, e, and f). The protein levels of α1 subunits (Ca_v_1.2 and Ca_v_1.3) and auxiliary β subunits (β_1–4_) and α_2_/δ1 subunit of L-type VGCCs in the PFC and hippocampus were not altered in Ahnak KO mice compared to WT mice (Supplementary Figure 3), suggesting that the reduced L-type calcium current is not due to decreased levels of the channels. We also found that the voltage-gated total calcium current and L-type-specific calcium current, but not non-L-type calcium current, were reduced in pyramidal neurons from the PFC of p11 KO mice (Supplementary Figure 4). Approximately 50% of the L-type calcium current was reduced in Ahnak or p11 KO neurons compared to WT controls, suggesting that Ahnak and p11 are critical in the regulation of L-type VGCCs in the brain.

### Ahnak plays an opposing role in excitatory versus inhibitory interneurons to regulate depressive behavior

**Fig. 5:**
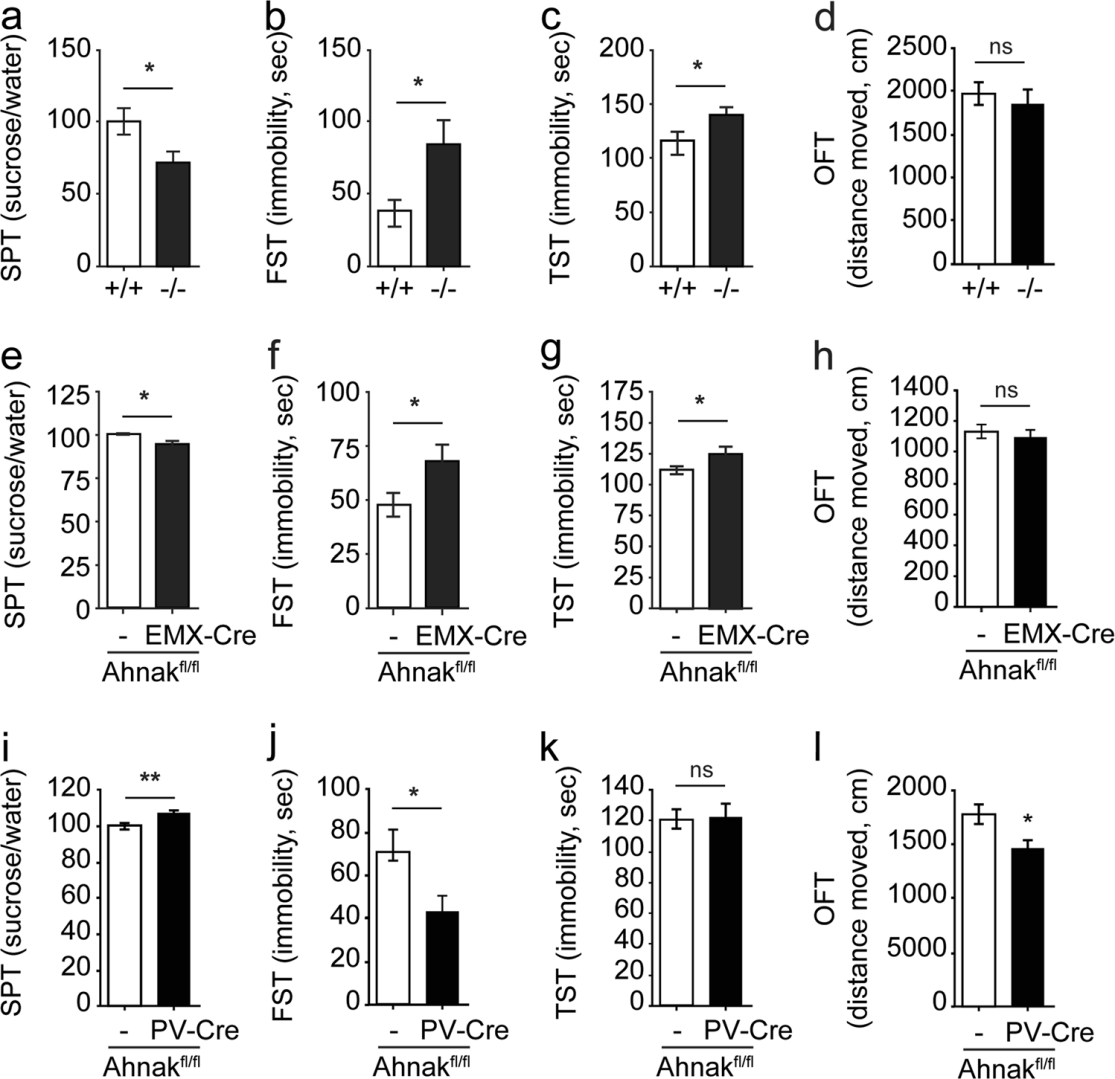
Behavioral phenotypes of constitutive or cell-type-specific Ahnak KO mice. Behavioral tests were performed with WT ( + / + ) and constitutive Ahnak KO (−/−) mice (**a-d**), forebrain glutamatergic neuron-specific Ahnak KO (Ahnak^f/f^ and EMX-Cre-positive^Cre/+^) and their control mice (Ahnak^f/f^, EMX-Cre-negative) (**e**-**h**) and PV-positive interneuron-specific Ahnak KO (Ahnak^f/f^ and PV-Cre-positive^Cre/+^) and their control mice (Ahnak^f/f^, PV-Cre-negative) (**i–l**). Sucrose preference test (SPT) (**a, e, i**), forced swim test (FST) (**b, f, j**), tail suspension test (TST) (**c, g, k**), and open field test (OFT, 10 min) (**d**, **h**, **l**). The sucrose preference was normalized to the value of WT (or control) group as 100. *n* = 14 (**a**), 10 (**b**), 12 (**c**), 15 (**d**), 20 (**e–h** and **k**), or 19 (**l**) per group; *n* = 20 (control) and 18 (PV-KO) (**i**); *n* = 19 (control) and 16 (PV-KO) (**j**). *p < 0.05, **p < 0.01, ns: non-significant, unpaired two-tailed *t* test. All bar graphs are means ± SEM

Previous studies have shown that constitutive p11 KO mice display a depression-like behavioral phenotype [3], while constitutive heterozygote Ca_v_1.2 KO [55, 56] or homozygote Ca_v_1.3 KO [57] caused an antidepressant-like behavioral phenotype. Given the critical roles for Ahnak in scaffolding and stabilizing p11 and Anxa2 proteins and in regulating L-type VGCCs, we examined whether the loss of Ahnak results in depression-like or antidepressant-like behaviors by measuring depression-related behaviors. Anhedonia can be modeled in rodents by measuring preference for a palatable sucrose solution [58]. Our behavioral analyses revealed that constitutive Ahnak KO mice have reduced preference for sucrose compared to WT littermate controls in the SPT (Fig. 5a). We also performed the FST and the TST, both of which measure behavioral immobility in response to an inescapable stressor [58]. Ahnak KO mice displayed increased immobility in the FST (Fig. 5b) and TST (Fig. 5c). However, Ahnak KO mice did not show altered motor activity compared to WT mice as measured by total distance traveled in the OFT (Fig. 5d and Supplementary Figure 6a). Altogether, constitutive Ahnak KO mice mimic the anhedonic and depression-like phenotype observed in the constitutive p11 KO mice [3], supporting Ahnak as a critical component of the p11 protein complex regulating depressive behavior.

Since Ahnak regulates L-type VGCCs in glutamatergic neurons as well as PV GABAergic interneurons, we generated forebrain glutamatergic neuron-specific or PV-specific Ahnak KO mice by crossing floxed Ahnak mice with EMX-Cre mice or PV-Cre mice, respectively (Supplementary Figure 5a). Despite prominent expression of Ahnak in vascular structures [13] (Fig. 2 and Supplementary Figure 2), forebrain glutamatergic neuron-specific Ahnak KO decreased the total Ahnak protein level by ~70% in the hippocampus and ~55% in the PFC, indicating that neuronal expression constitutes a major portion of Ahnak level in the brain (Supplementary Figure 5b). In contrast, because PV-positive GABAergic interneurons constitute a minor portion of cells in the brain, PV-positive GABAergic interneuron-specific Ahnak KO caused a negligible effect in total protein level of Ahnak in the hippocampus and PFC (Supplementary Figure 5c).

Importantly, forebrain glutamatergic neuron-specific Ahnak KO mice displayed depression-like behaviors in SPT, FST, and TST (Fig. 5e-g) in the absence of significant change in motor activity (Fig. 5h and Supplementary Figure 6b). In contrast, PV-specific Ahnak KO mice displayed antidepressant-like behaviors in SPT and FST, but not in TST (Fig. 5i-k). A moderate hypoactive phenotype restricted to the initial 10 min in OFT (Fig. 5l and Supplementary Figure 6c) might have obscured an antidepressant-like effect of the PV-specific Ahnak KO mice in the TST (Fig. 5k). However, overall motor activity (for 60 min) of the PV-KO mice in OFT was comparable to their control mice (Supplementary Figure 6c). Altogether these results indicate that Ahnak plays opposing roles in excitatory versus inhibitory neurons to regulate depressive behavior.

## Discussion

In this study, we demonstrated that Ahnak, as a major binding partner of the p11/Anxa2 protein complex, stabilizes p11 and Anxa2 proteins in the brain. Previously, the specific interaction between Ahnak and the p11/Anxa2 complex was characterized in cell lines, yeast triple-hybrid assays and in vitro experiments [12, 59]. In addition, our previous structural study clearly demonstrated the molecular interaction between Ahnak and the p11/Anxa2 complex at the atomic level [9]. Thus, the results from the current study, together with previous reports, highly suggest the existence of an endogenous Ahnak/p11/Anxa2 complex in the brain. In line with this notion, constitutive Ahnak KO mice mimic the depression-like phenotype seen in constitutive p11 KO mice. We also showed that Ahnak plays an opposing role in excitatory neurons versus inhibitory interneurons to regulate depression-like behaviors, a finding which is similar to the roles for p11 and its binding partner, metabotropic glutamate receptor 5 (mGluR5), observed in our previous study [43].

Previous studies indicate that p11 interacts with various membrane receptors and ion channels. p11 binds to several subtypes of serotonin receptors (5-HT1B, 1D, and 4 receptors) [3, 7] and increases serotonergic neurotransmission by increasing cell surface expression of serotonin receptors [2]. Additionally, p11 binds to mGluR5 and increases its cell surface availability [43]. p11 also interacts with several ion channels, such as TASK-1 (a 2 P domain K^+^ channel family protein) [60], TRPV5 and 6 (transient receptor potential channels) [61], and Nav1.8 (a sodium channel) [62] and is involved in their trafficking to the cell surface. The roles of p11 in the accumulation of these binding proteins at the plasma membrane bear resemblance to the role of Ahnak in the cell surface expression of Ca_v_1.2 or Ca_v_1.3. Thus, Ahnak may be involved in the interaction with and trafficking of some of these p11-binding ion channels and membrane receptors.

Our study has also demonstrated that Ahnak functionally scaffolds L-type VGCCs as a downstream effector of the Ahnak/p11/Anxa2 complex. Ahnak KO causes a decrease of cell surface levels of Ca_v_1.2 and Ca_v_1.3, resulting in a decrease of voltage-induced L-type-specific calcium influx. Previous studies with cardiomyocytes suggested that the C-terminal region of Ahnak interacts with the cytoplasmic auxiliary β2 subunit of VGCCs [26, 49]. In this study, we also observed an interaction between the C-terminal fragment of Ahnak and the β4b subunit. Notably, β subunits promiscuously bind to all high-voltage-activated calcium channels including L-, N-, R- and P/Q type VGCCs [63]. Thus, interaction of Ahnak with the L-type α1 subunit, rather than the promiscuous β subunit, may explain the L-type-specific regulation by Ahnak observed in this study.

L-type calcium channel activity is significantly reduced in both excitatory and inhibitory neurons in Ahnak KO mice, and L-type calcium channel influx is known to regulate neuronal excitability [19, 64, 65]. Thus, the opposing behavioral phenotype is presumably caused by a decrease or an increase of the excitation/inhibition (E/I) ratio in neural circuitry. This notion is consistent with models proposed for depressive- or antidepressant-like behavior caused by p11 KO or mGluR5 KO [43] and fast-acting antidepressant-like effects of an mGluR5 antagonist [43] or ketamine [66, 67]. In fact, recent studies of conditional KO or knockdown of Ca_v_1.2 indicate that L-type VGCCs may have opposing roles in depression-like behavior or stress susceptibility, depending on the neuronal circuits, brain regions, or the developmental stages [56, 68, 69]. Thus, cell-type-specific and circuit-based investigation of Ahnak is necessary to fully understand its role in the control of depressive behavior.

Human genetic studies implicate altered function of L-type VGCCs in the pathophysiology of multiple psychiatric disorders including MDD, bipolar disorder, schizophrenia, and autism spectrum disorder [22–24, 70–72]. Notably, imbalance of the E/I ratio is also implicated in the pathophysiology of schizophrenia and autism spectrum disorder [73–75]. Thus, testing whether alteration of Ahnak in specific neuronal classes causes other behavioral abnormalities relevant to psychiatric disorders is another important subject for future studies.

Ahnak seems to be a multifunctional protein in the brain. In addition to neurons, Ahnak is markedly expressed in endothelial cells in vascular structures, in epithelial cells in choroid plexus, and in ependymal cells in the ventricular walls in the brain [13, 14]. Ahnak expression level and localization were highly correlated with tight junction formation of endothelial cells in the blood–brain barrier and of epithelial cells in the choroid plexus [13]. A potential role for Ahnak in blood–brain barrier function and the potential impact of such an effect on behaviors relevant to psychiatric disorders remain to be investigated.

In conclusion, our current study suggests the Ahnak/p11/Anxa2 complex as an endogenous neuronal machinery controlling cell surface expression of L-type VGCCs and thereby scaling neuronal activity-induced calcium signaling. Constitutive Ahnak KO mice display depression-like behavior, but Ahnak plays an opposing role in excitatory neurons versus inhibitory interneurons to regulate depressive behavior. To translate our findings into clinical relevance, potential alterations of molecular components of this novel pathway should be investigated in mouse models of depression as well as brains of depressed patients. The current and future studies of Ahnak and its molecular complex will contribute not only to our understanding of the pathophysiology of depression but also to the development of novel molecular targets for therapeutics.

## Supplementary information

Supplementary Figure 1

Supplementary Figure 2

Supplementary Figure 3

Supplementary Figure 4

Supplementary Figure 5

Supplementary Figure 6
