# Supplementary material for: Ahnak scaffolds p11/Anxa2 complex and L-type voltage-gated calcium channel and modulates depressive behavior
Source: Mol Psychiatry. 2019 Feb 13;25(5):1035–49. doi: 10.1038/s41380-019-0371-y (PMC6692256; doi:10.1038/s41380-019-0371-y)
Supplement: Supplementary file 1 — Supplementary Figure 1 [file 41380_2019_371_MOESM1_ESM.docx]

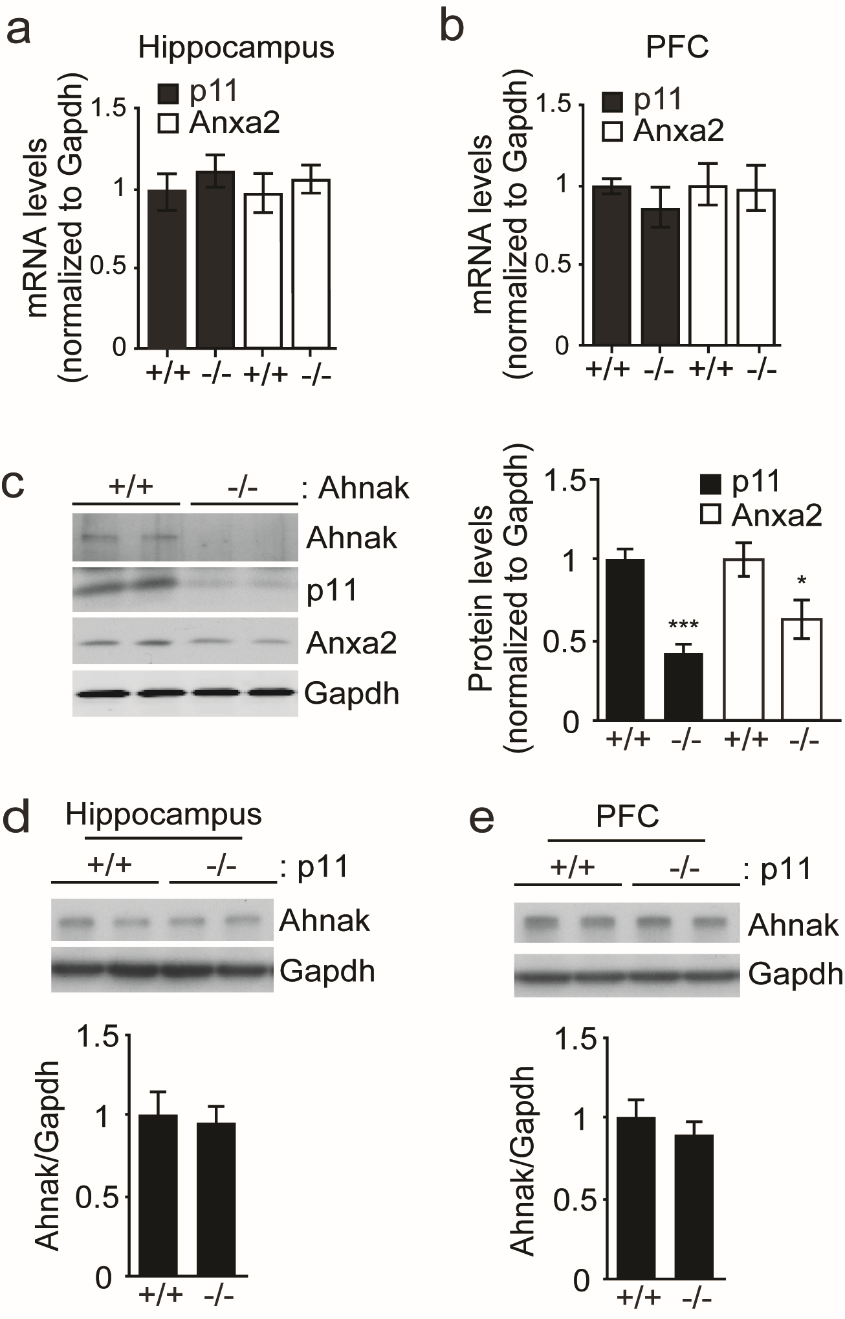


**Supplementary Figure 1**. mRNA levels of p11 and Anxa2 in Ahnak KO brain tissues, protein levels of p11 and Anxa2 in primary cultures of Ahnak KO neurons, and Ahnak protein level in p11 KO brains. (**a, b**) mRNA levels of p11 (**a**, n=12 for +/+ and 11 for -/-; **b**, n=16 for +/+ and 14 for -/-) and Anxa2 (**a**, n=10 for both groups; **b**, n=8 for +/+ and 7 for -/-) were measured by qPCR. mRNA levels were not altered in the hippocampus (**a**) and PFC (**b**) of Ahnak KO mice compared to WT mice. (**c**) Protein levels of p11 and Anxa2 in primary cultured cortical neurons prepared from WT or Ahnak KO embryos. Representative images of immunoblotting and quantification of protein levels are shown (n=5 for +/+ and 6 for -/-). **p*<0.05, ****p*<0.001, *t* test. (**d**, **e**) Protein levels of Ahnak and Gapdh as a loading control in the hippocampus (**d**) or PFC (**e**) of WT (+/+) and Ahnak KO (-/-) mice were analyzed. Representative images and quantification of protein levels (n=8 per group) are shown. All bar graphs are means ± SEM.
