# Supplementary material for: Ahnak scaffolds p11/Anxa2 complex and L-type voltage-gated calcium channel and modulates depressive behavior
Source: Mol Psychiatry. 2019 Feb 13;25(5):1035–49. doi: 10.1038/s41380-019-0371-y (PMC6692256; doi:10.1038/s41380-019-0371-y)
Supplement: Supplementary file 2 — Supplementary Figure 2 [file 41380_2019_371_MOESM2_ESM.docx]

**
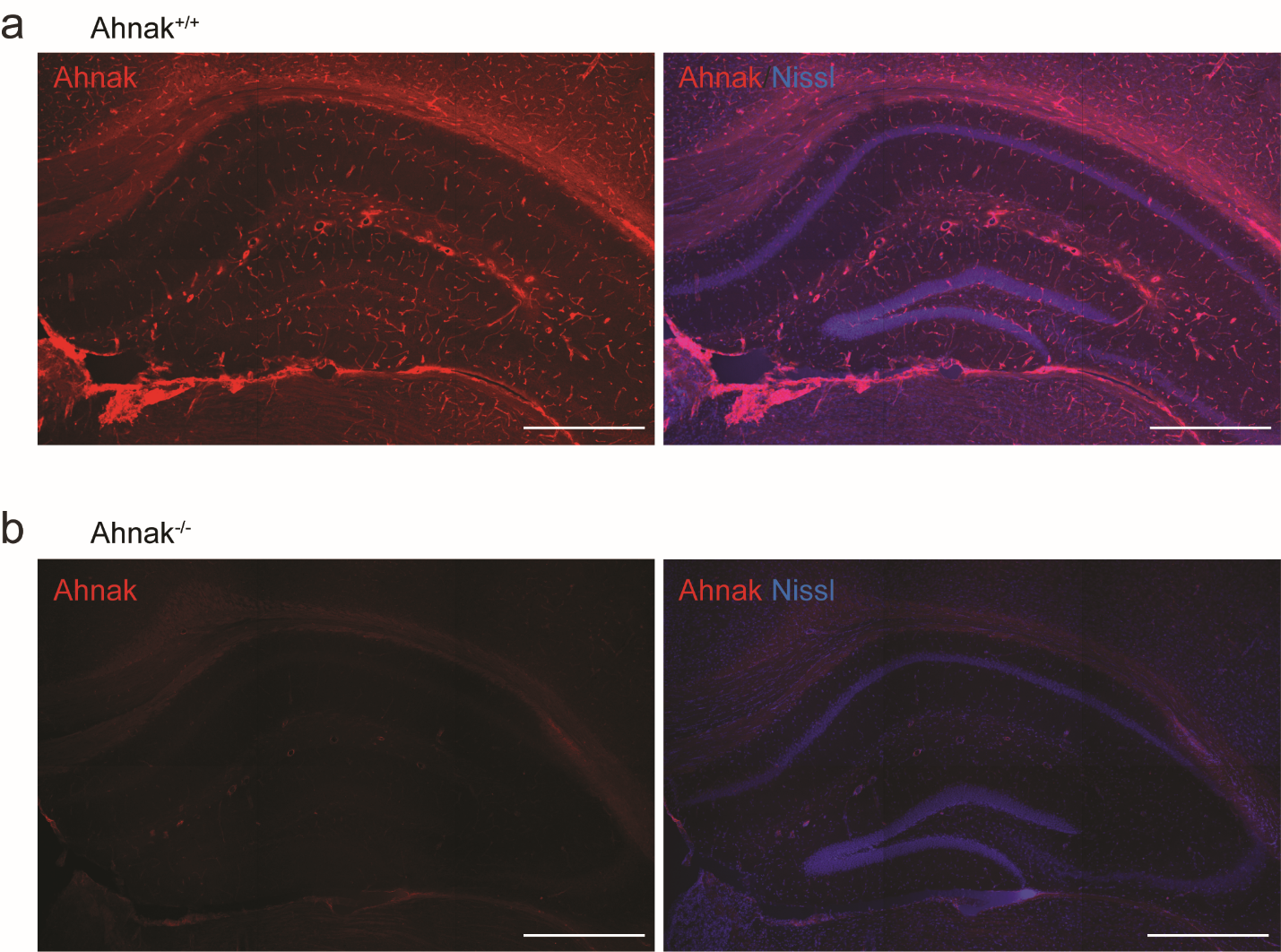
**

**Supplementary Figure 2**. Specificity of anti-Ahnak antibody for immunohistochemistry. Hippocampal brain slices from WT (**a**) and Ahnak KO mice (**b**) were subjected to immunohistochemistry with anti-Ahnak antibody. Scale bars, 500 µm. Nissl counterstaining was used to visualize neural anatomy.
