# Supplementary material for: Ahnak scaffolds p11/Anxa2 complex and L-type voltage-gated calcium channel and modulates depressive behavior
Source: Mol Psychiatry. 2019 Feb 13;25(5):1035–49. doi: 10.1038/s41380-019-0371-y (PMC6692256; doi:10.1038/s41380-019-0371-y)
Supplement: Supplementary file 3 — Supplementary Figure 3 [file 41380_2019_371_MOESM3_ESM.docx]

**
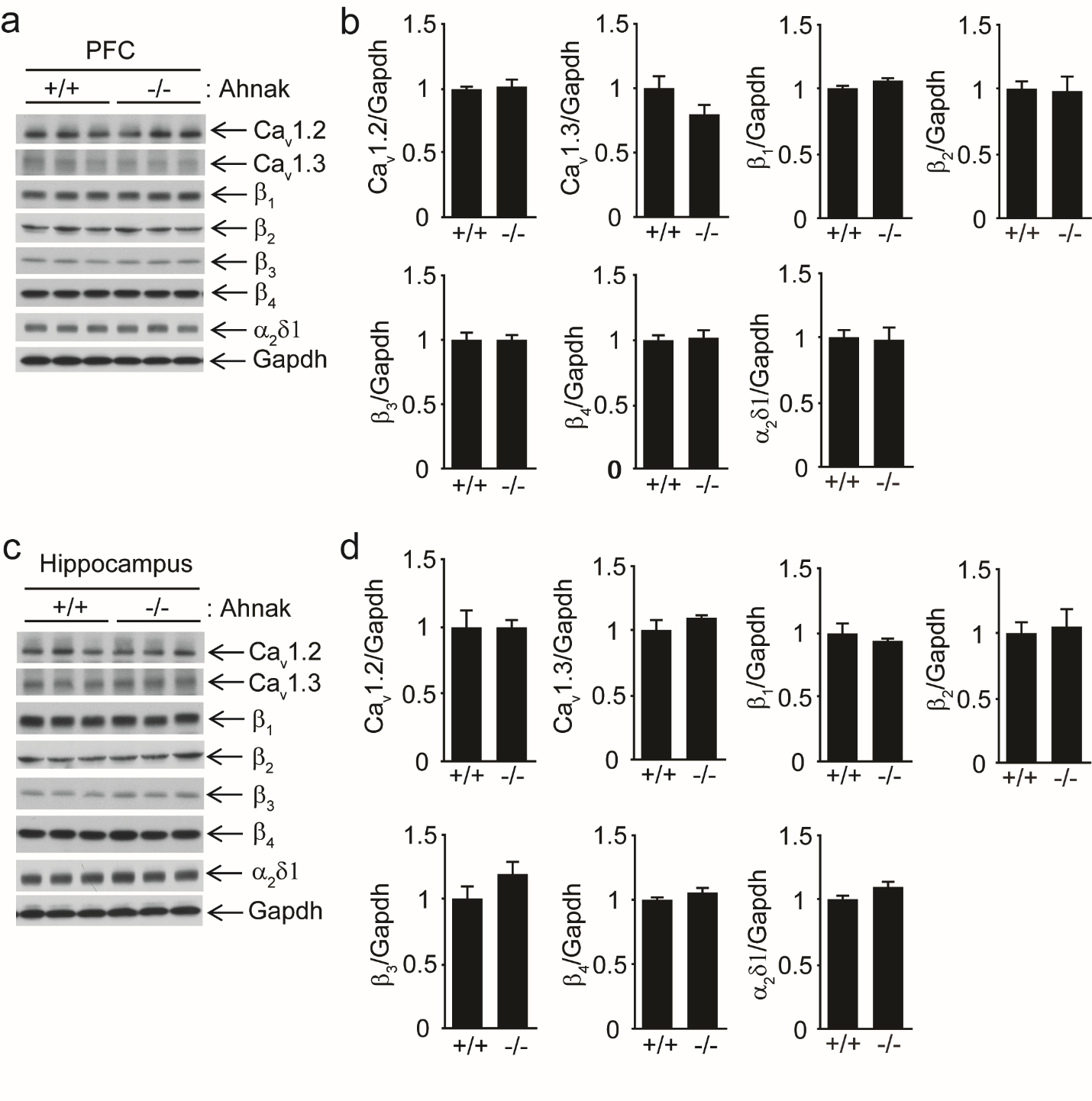
**

**Supplementary Figure 3**. Protein levels of subunits of L-type VGCCs were not altered in Ahnak KO mice compared to WT mice. Protein levels of two α1 subunits, four β subunits, α_2_δ1 subunit and Gapdh in the PFC (**a**, **b**) and hippocampus (**c**, **d**) were analysed by immunoblotting. Representative images of immunoblotting (**a**, **c**) and quantification of protein levels (n=6 per group) (**b**, **d**) are shown. Bars are means ± SEM.
