# Supplementary material for: Ahnak scaffolds p11/Anxa2 complex and L-type voltage-gated calcium channel and modulates depressive behavior
Source: Mol Psychiatry. 2019 Feb 13;25(5):1035–49. doi: 10.1038/s41380-019-0371-y (PMC6692256; doi:10.1038/s41380-019-0371-y)
Supplement: Supplementary file 4 — Supplementary Figure 4 [file 41380_2019_371_MOESM4_ESM.docx]

**
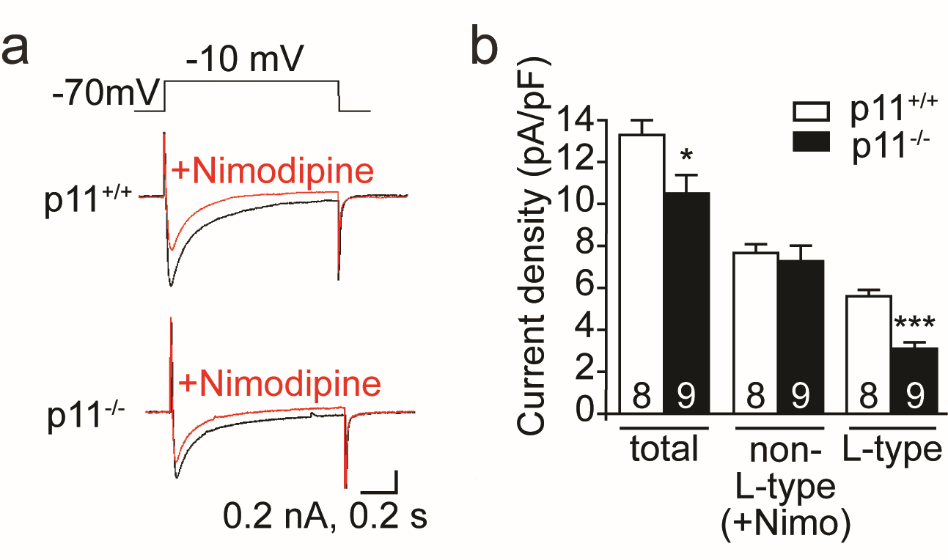
**

**Supplementary Figure 4**. L-type-specific calcium current is reduced in p11 KO neurons. (**a**, **b**) Whole-cell patch-clamp recording of voltage-dependent Ca^2+^ current in pyramidal neurons in layer 2/3 of PFC from WT and p11 KO mice. Somatic Ca^2+^ currents were recorded before and after the bath application of an L-type calcium current blocker (nimodipine). Representative traces (**a**). Histograms showing the density of total, L-type and non-L-type voltage-dependent Ca^2+^ currents in neurons from WT and p11 KO mice (**b**). Bar graphs are means ± SEM. **p*< 0.05, ****p*< 0.001, student’s *t* test. Numbers of neurons used for recording are indicated in each bar. 3-5 mice per group.
