# Supplementary material for: Ahnak scaffolds p11/Anxa2 complex and L-type voltage-gated calcium channel and modulates depressive behavior
Source: Mol Psychiatry. 2019 Feb 13;25(5):1035–49. doi: 10.1038/s41380-019-0371-y (PMC6692256; doi:10.1038/s41380-019-0371-y)
Supplement: Supplementary file 5 — Supplementary Figure 5 [file 41380_2019_371_MOESM5_ESM.docx]

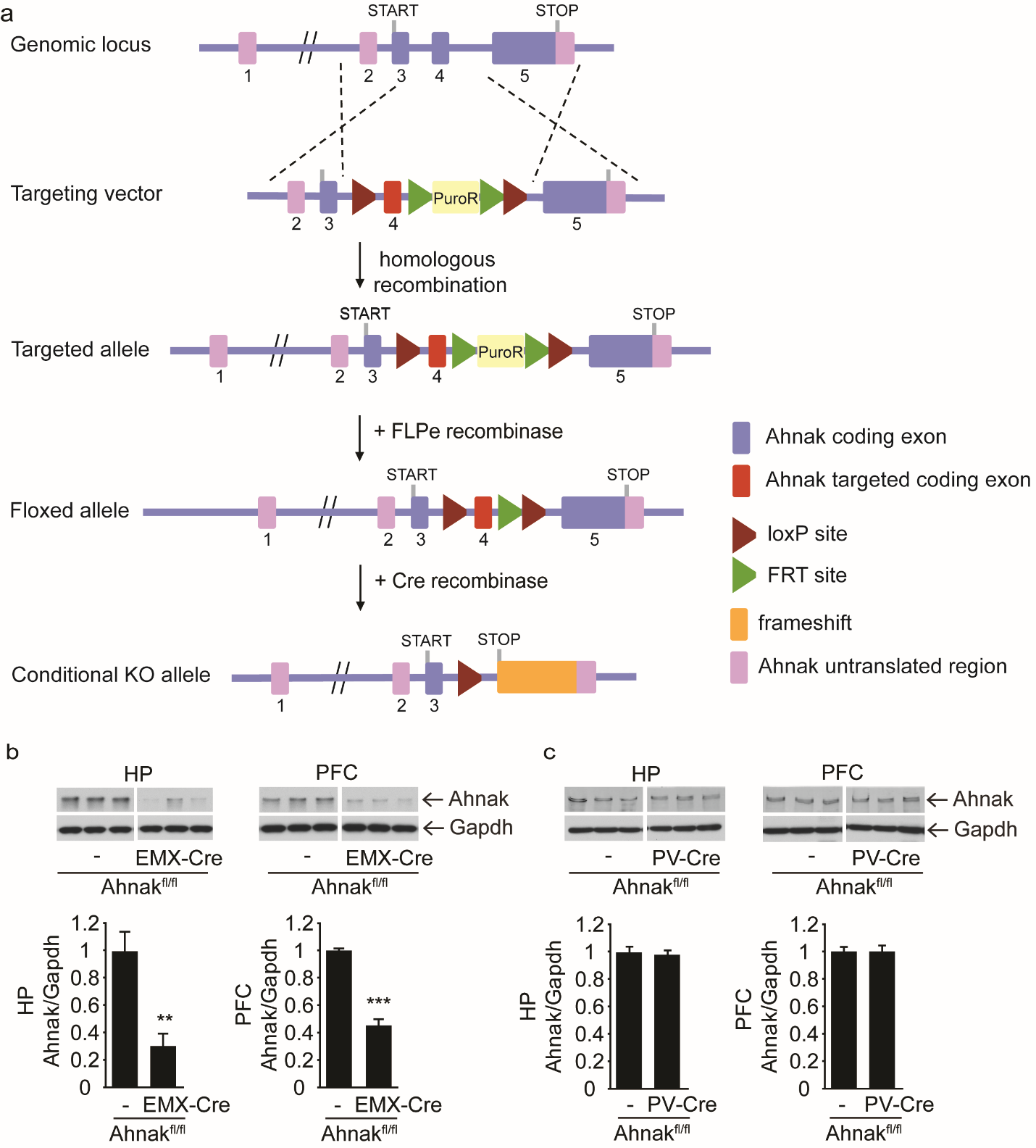


**Supplementary Figure 5**. Generation of cell type-specific Ahnak KO mice. (**a**) Schematic for Ahnak gene KO strategy. In the targeting vector, exon 4 was flanked by loxP sites. A selection marker (Puromycin resistance-Puro R) flanked by FRT sites was inserted downstream of exon 4. The selection marker in the targeted allele was deleted after breeding with FLP mice expressing efficient FLP recombinase. Forebrain glutamatergic-specific Ahnak KO mice were generated by breeding floxed Ahnak mice with EMX-Cre recombinase mice. Deletion of exon 4 creates a frame shift in downstream exons and generates an early STOP codon. (**b, c**) Alteration of Ahnak protein in EMX-Cre-mediated Ahnak KO brains (**b**) or PV-Cre-mediated Ahnak KO brains (**c**). Protein levels of Ahnak and Gapdh in the hippocampus (HP, left) or PFC (right) of WT and Ahnak KO mice were analysed by immunoblotting (n=7 per group). All bar graphs are means ± SEM. **p<0.01, ***p<0.001, t test.
