# Supplementary material for: Ahnak scaffolds p11/Anxa2 complex and L-type voltage-gated calcium channel and modulates depressive behavior
Source: Mol Psychiatry. 2019 Feb 13;25(5):1035–49. doi: 10.1038/s41380-019-0371-y (PMC6692256; doi:10.1038/s41380-019-0371-y)
Supplement: Supplementary file 6 — Supplementary Figure 6 [file 41380_2019_371_MOESM6_ESM.docx]

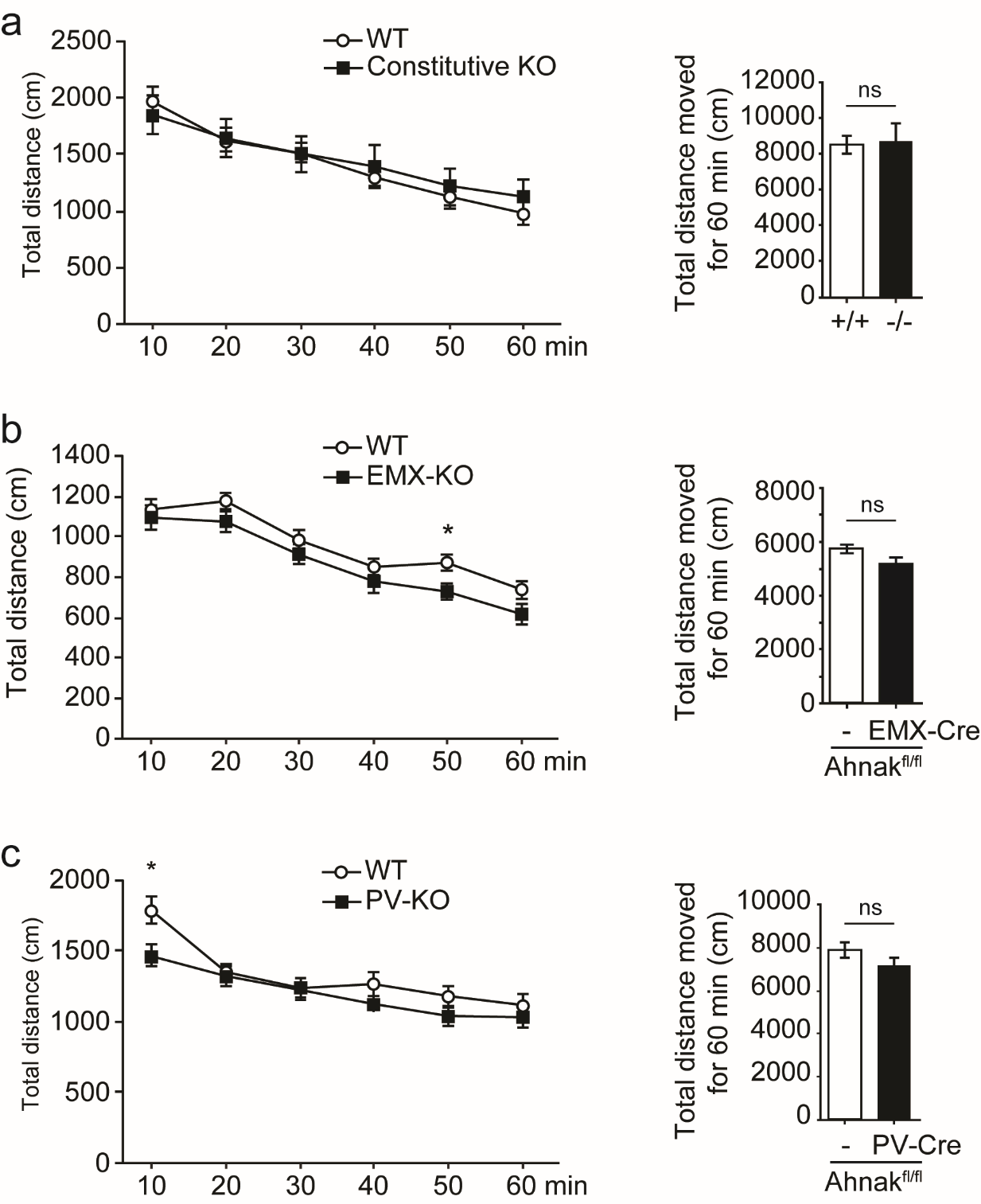


**Supplementary Figure 6**. Motor activities of constitutive Ahnak KO mice and cell-type-specific Ahnak KO mice were measured in open field tests. (**a**) Constitutive Ahnak KO mice and WT mice. (**b**) Forebrain glutamatergic neuron-specific Ahnak KO (Ahnak^f/f^ and EMX-Cre-positive^Cre/+^, EMX-KO) and their control mice (Ahnak^f/f^ and EMX-Cre-negative, WT) and (**c**) PV-positive interneuron-specific Ahnak KO (Ahnak^f/f^ and PV-Cre-positive^Cre/+^) and their control mice (Ahnak^f/f^ and PV-Cre-negative, WT). Total distance travelled was shown with each 10 min-bin (left). The bar graphs indicate total distance travelled for 60 min (right). All graphs are means ± SEM. *p<0.05, two-way ANOVA, Bonferroni’s post hoc test.
